# Supplementary material for: Genetic evidence of a hybrid swarm between Alpine ibex (Capra ibex) and domestic goat (C. hircus)
Source: Evol Appl. 2024 Jul 28;17(8):e13761. doi: 10.1111/eva.13761 (PMC11284124; doi:10.1111/eva.13761)
Supplement: Supplementary file 1 — Appendix S1. [file EVA-17-e13761-s001.docx]

Supplementary Material

Genetic evidence of a hybrid swarm between Alpine ibex (*Capra ibex*) and domestic goat (*C. hircus*)

Alice Brambilla, Noel Zehnder, Bruno Bassano, Luca Rossi, Christine Grossen

**Supplementary Figures**

**
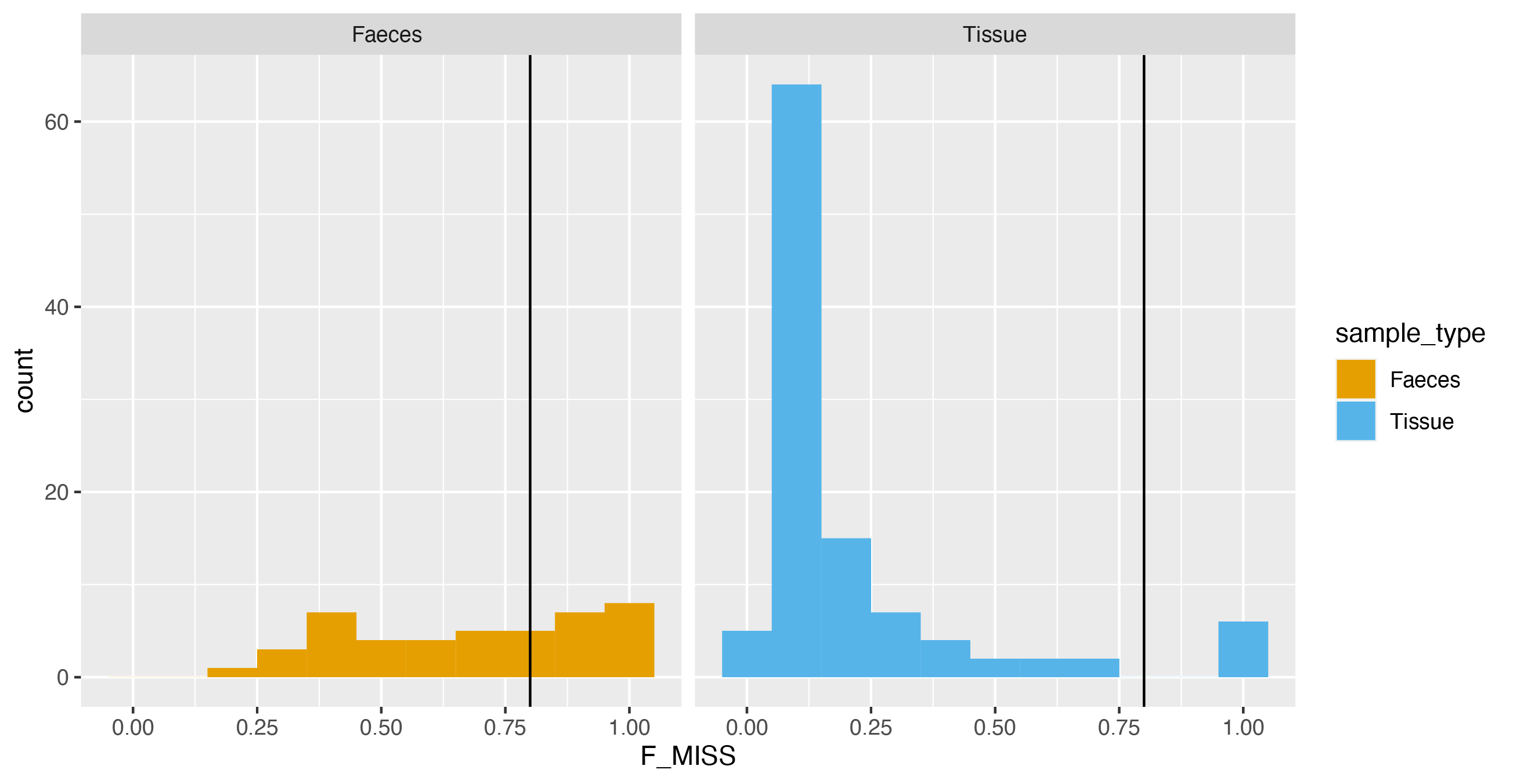
**

**Supplementary Figure S1: Number of samples of different kind (faeces in yellow and tissue in light blue) with different proportion of missingness (F_MISS). Black line represents the threshold used in this study to filter out samples with high missingness (80%).**

**
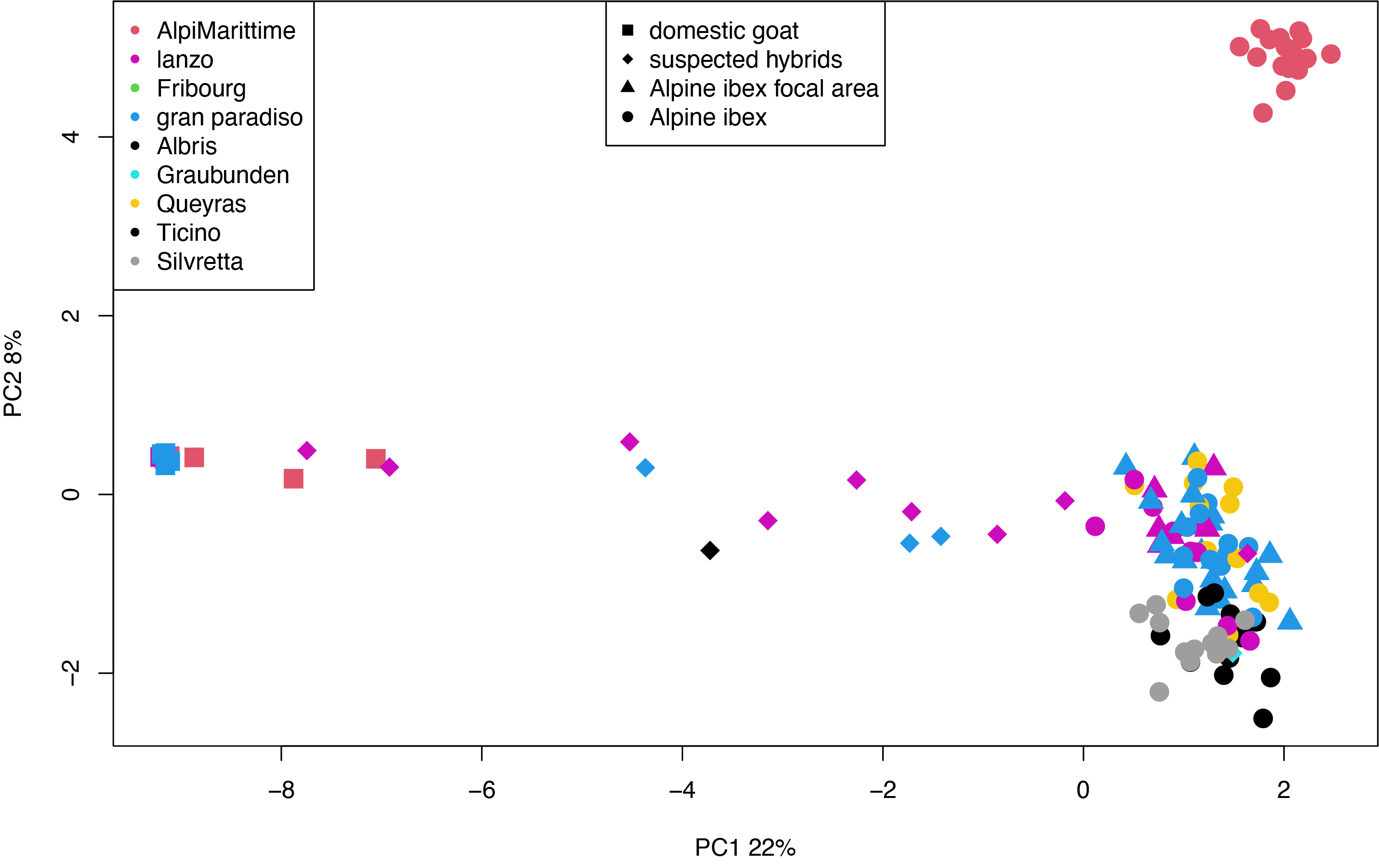
**

**Supplementary Figure S2:** Principal component analysis (PCA) based on 465 neutral SNPs on 133 individuals. Colours indicate different Alpine ibex populations. Shapes indicate the category of the individuals included (dot: non-suspected Alpine ibex outside the focal area; triangle: non-suspected Alpine ibex in the focal area; rhombus; suspected hybrids; square: non-suspected domestic goats.


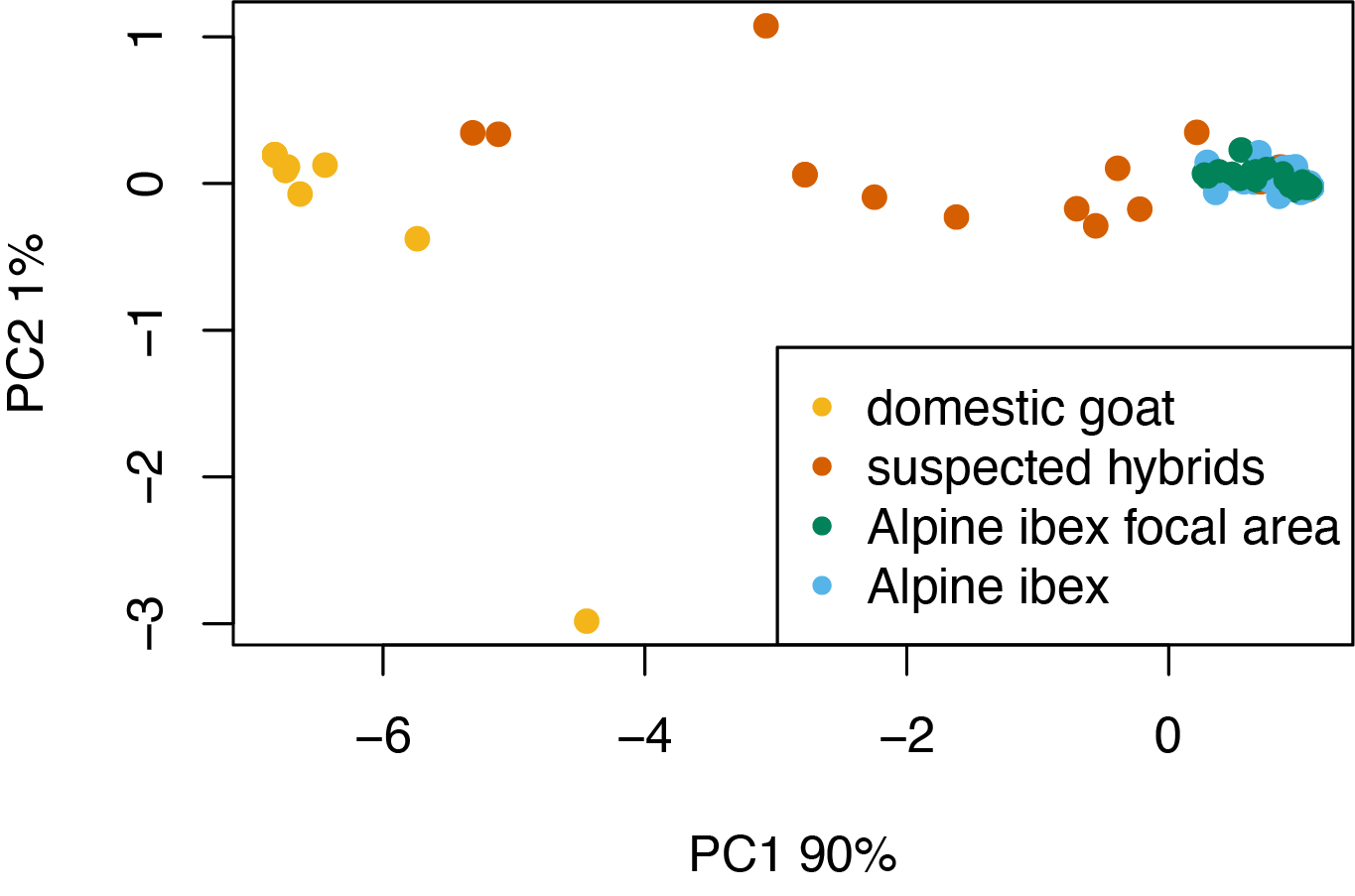


**Supplementary Figure S3:** Principal component analysis (PCA) on 133 individuals based on 63 SNPs diagnostic for the comparison Alpine ibex versus domestic goat. Colours as in Figure 1.

**Supplementary Figure S4:** Cumulative probabilities computed by NewHybrids for each individual to belong to one of the pre- defined hybrid classes. The plot includes visualization of all tested categories. Categories likely occurring at the same generation were merged in the main figure (Fig, 2A) to improve readability. See Table **S4 for the custom genotype frequencies used for each category in the NewHybrids analysis and Table S6 for the posterior probabilities estimated by NewHybrids.**

**Abbreviations: Goat: domestic goat, Ibex: Alpine ibex, F1: F1 hybrid, F2: mating between two F1 hybrids, BxG: mating between F1 and domestic goat (back-cross into domestic goat), BxI: mating between F1 and Alpine ibex (back-cross into Alpine ibex), BxGxG: mating between BxG and domestic goat (back-cross 2^nd^ generation into domestic goat), BxIxI: mating between BxI and Alpine ibex (back-cross 2^nd^ generation into Alpine ibex), BxGxI: mating between BxG and Alpine ibex, BxIxG: mating between BxI and domestic goat, F1xBxG: mating between F1 and BxG, F1xBxI: mating between F1 and BxI, BxGxGxG: back-cross 3^rd^ generation into domestic goat, BxIxIxI: back-cross 3^rd^ generation into Alpine ibex, BxGxBxG: mating between two back-cross into domestic goat, BxIxBxG: mating between back-cross into Alpine ibex and back-cross into domestic goat.**

**
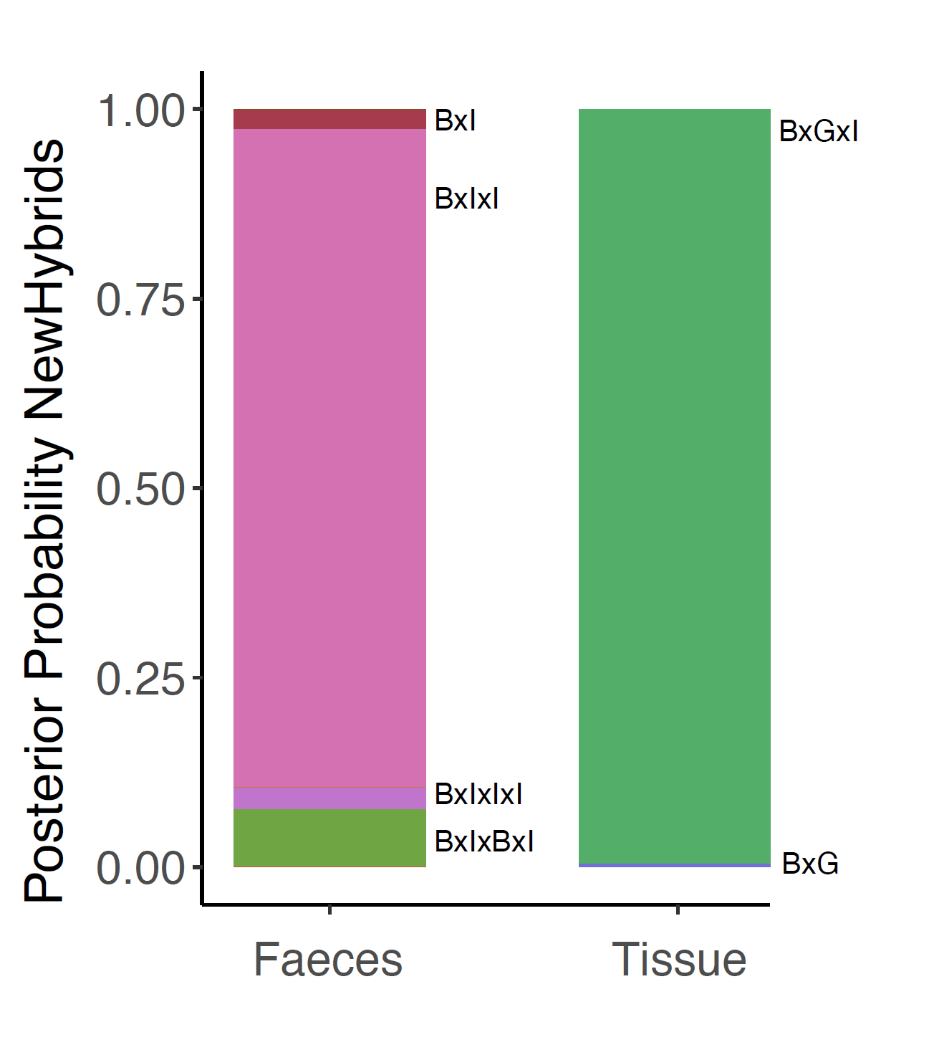
**

**Supplementary Figure S5:** Cumulative probabilities computed by NewHybrids for the two samples (tissue and faecal) collected from the same individual to belong to one of the pre- defined hybrid classes. The plot includes visualization of all tested categories. Abbreviations as in Figure S4.

**Supplementary Tables**

**Supplementary Table S1**: Description and pictures of the typical phenotype of the Alpine ibex (based on Couturier, 1962 and Brambilla et al., 2020) and of the local breed of domestic goat (based on the description provided by the Association Régionale Eleveurs Valdôtains (AREV) available at: <https://www.arev.it/allevamento/razze-caprine>). The breeds phenotypically closest to Alpine ibex and widespread in the study region are the *Capra camosciata delle Alpi* and the *Capra Valdostana*, a local variety of *Camosciata delle Alpi* mainly present in the Valle d’Aosta region (Italy).

| Trait | Alpine ibex (*Capra ibex*) | Domestic goat (*Capra hircus*), *Valdostana* breed |
| --- | --- | --- |
| Picture | 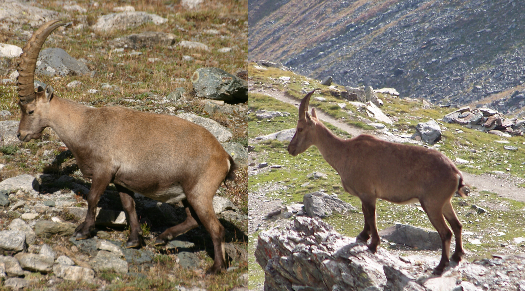 | 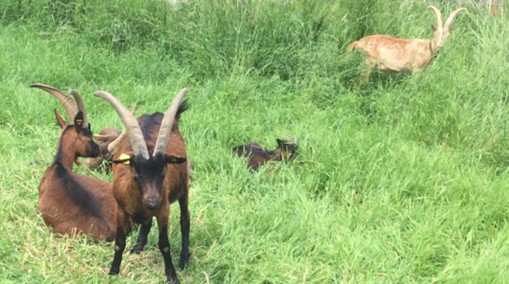 |
| Body | Compact, with short and broad head and neck. Due to shorter metapodials, legs are also relatively short and robust compared to other ungulates. Males are larger and heavier than females: they can grow up to 163 cm in length and 94-95 cm at withers. Females are smaller with a body length of 127–139 cm and height at withers of 81–82 cm. Body mass of fully grown males is around 75–95 kg (with a maximum up to 130 kg) while that of females ranges instead between 35 and 45 kg (max. up to 60 kg). Straight back with less prominent kyphosis (compared to domestic goats).  Females have a single pair of udders, rarely hanging even during lactation. | Medium-large body size compared to other goat breeds in the Alps but smaller than Alpine ibex (height at withers 70-80 cm, maximum body mass 65 kg in females and 85 kg in males).  Well-developed udders, obviously hanging during lactation. |
| Horn | Horns of both sexes are backward-curving and sabre-shaped, grow lifelong and develop an annual ring during each winter. Male horns are longer (up to 100 cm in old individuals) and thicker than those of females (which only exceptionally are longer than 35 cm). The cross section of the horns is rectangular in males (with 5-7 to 25-30 cm circumference) and oval in females (4-5 to 12-15 cm circumference). Male horns are also characterized by typical pronounced nodes on the frontal side (up to three per year) which are less pronounced in females.  Well-marked annual rings. | Exceptionally long compared to other goat breeds but seldom longer than 50 cm. Sabre-shaped, backward curving, with triangular cross section, usually with smooth surface. If present, nodes are less pronounced and more flattened than those of Alpine ibex. Poorly marked to absent annual rings. |
| Muzzle | Large and thick with a convex shape due to the frontal bone. Ears are small with rounded tips. Eyes are yellowish with a horizontal, squared, pupil. | Relatively small head, with snub/concave profile, broad forehead. |
| Pelage | Short, straight and strong hairs. The coat is rather uniform in appearance with brownish-grey hair over most of the body. The abdomen, the rump patch, the hairy part of the tail bottom as well as the caudal part of the tarsus can be paler, particularly in females and kids, but the general aspect is uniform. In autumn coat is complemented with longer and darker top hairs and a dense, woolly undercoat. Coats vary in colour with sex and age from brownish grey to reddish chestnut brown in summer to brown-grey in winter. Newborns are pale brown. Adult males may have a short chin beard. | Epidermis is usually black, four main coats are typical of the breed: 1) chestnut brown with melanic areas (legs, head), often with a dark crest along the back and on the tail; 2) uniform black; 3) uniform grey-blond; 4) dark coat with blond hair tips. |

**Supplementary Table S2**: Description of the anomalies, compared to the wild Alpine ibex phenotype (described in Supplementary Table S1), reported in literature (^1^Couturier, 1962; ^2^Giacometti et al., 2004; ^3^Moroni et al., 2022) and observed in our study in the diagnostic traits used to identify the suspected hybrids. Unless differently specified, the characteristics refers to both males and females. Some of the anomalies observed in our study were not reported in literature but deviate from the Alpine ibex phenotype and are more typical of domestic goats.

| Trait | Anomalies reported in literature | Anomalies observed in the study (from confirmed hybrids) |
| --- | --- | --- |
| Pelage | Non uniform coat color^1^;  Coat colours: dark brown^1, 2, 3^, brownish or greyish with white undersides^2^;  Prominent leg markings (dark brown front, white back)^1^;  Lack of winter wool hair^1^, | Coat colours: black, dark brown, orange-yellow;  Prominent dark leg markings;  Dark legs and dark crest along the back;  Lack of winter wool hair |
| Horn | Absence of horns (ff^1^)^3^;  Longer horns^1,2^ (in males annual increments of 110-240% compared to Alpine ibex^2^);  Sharp-keeled horns laterally flattened (triangular cross section), with occasional flattened nodes^2^;  Horns with round cross-section and narrow frontal and caudal features^2^. | Absence of horns (female);  Longer annual increments;  Horns with triangular cross section;  Thinner horns;  Nodeless horns;  Horns with flattened nodes |
| Muzzle | Intermediate aspect between Alpine ibex and domestic goat^1^ | Broad forehead;  Concave profile;  Acute triangle profile;  Longer beard (male);  Longer ear pinnae |
| Body | Larger body size^2^ (in male 110% compared to same age Alpine ibex^2^);  Smaller body size^1^;  Saddled back, dorsal kyphosis, bony and protruding hips^1^ | Larger body size;  Protruding hips;  Visible udders (female);  Larger testicles (male) |

**Supplementary Table S3: Genomic positions of diagnostic markers used in the study Kessler et al. (2020) included 84 diagnostic markers but not all of them passed the filtering applied for this study).**

| Chromosome | Position (bp) |
| --- | --- |
| 1 | 5006951 |
| 1 | 20009171 |
| 1 | 115038611 |
| 2 | 25007919 |
| 2 | 85013828 |
| 2 | 100018140 |
| 2 | 120020686 |
| 3 | 45006553 |
| 3 | 60009115 |
| 4 | 5023209 |
| 4 | 20028817 |
| 4 | 40053795 |
| 4 | 70059490 |
| 5 | 10014831 |
| 5 | 40017267 |
| 5 | 55045427 |
| 5 | 70046599 |
| 5 | 115050605 |
| 6 | 25058595 |
| 6 | 70061342 |
| 7 | 20011419 |
| 7 | 75036297 |
| 7 | 95037984 |
| 8 | 45047108 |
| 8 | 60049021 |
| 9 | 70040547 |
| 10 | 24784 |
| 10 | 20025417 |
| 10 | 40031796 |
| 10 | 60038098 |
| 11 | 5012391 |
| 11 | 70046052 |
| 11 | 90049112 |
| 12 | 5214898 |
| 12 | 20315678 |
| 12 | 40316282 |
| 12 | 70322246 |
| 13 | 10048151 |
| 13 | 25050600 |
| 13 | 55051511 |
| 13 | 80055158 |
| 14 | 25021789 |
| 15 | 20007044 |
| 15 | 65019388 |
| 16 | 30024980 |
| 16 | 50040434 |
| 17 | 30011647 |
| 17 | 45031043 |
| 18 | 15003075 |
| 18 | 55007685 |
| 19 | 10023896 |
| 19 | 45029681 |
| 20 | 30009781 |
| 21 | 20010967 |
| 21 | 55013563 |
| 23 | 25083623 |
| 24 | 20020388 |
| 26 | 40126121 |
| 27 | 20015442 |
| 28 | 5008953 |
| 28 | 40015753 |
| 29 | 20004050 |
| 29 | 35008446 |

**Supplementary Table S4: Custom genotype frequencies table used for NewHybrids analysis. Abbreviations as in Figure S4.**

| 17 |  |  |  |  |
| --- | --- | --- | --- | --- |
| Pure_G | 1.0000 | 0.0000 | 0.0000 | 0.0000 |
| Pure_I | 0.0000 | 0.0000 | 0.0000 | 1.0000 |
| F1 | 0.0000 | 0.5000 | 0.5000 | 0.0000 |
| F2 | 0.2500 | 0.2500 | 0.2500 | 0.2500 |
| BxG | 0.5000 | 0.2500 | 0.2500 | 0.0000 |
| BxI | 0.0000 | 0.2500 | 0.2500 | 0.5000 |
| BxGxG | 0.7500 | 0.1250 | 0.1250 | 0.0000 |
| BxIxI | 0.0000 | 0.1250 | 0.1250 | 0.7500 |
| BxGxI | 0.0000 | 0.3750 | 0.3750 | 0.2500 |
| BxIxG | 0.2500 | 0.3750 | 0.3750 | 0.0000 |
| F1xBxG | 0.3750 | 0.2500 | 0.2500 | 0.1250 |
| F1xBxI | 0.1250 | 0.2500 | 0.2500 | 0.3750 |
| BxGxGxG | 0.8750 | 0.0625 | 0.0625 | 0.0000 |
| BxIxIxI | 0.0000 | 0.0625 | 0.0625 | 0.8750 |
| BxIxBxI | 0.0625 | 0.1875 | 0.1875 | 0.5625 |
| BxGxBxG | 0.5625 | 0.1875 | 0.1875 | 0.0625 |
| BxIxBxG | 0.1875 | 0.3125 | 0.3125 | 0.1875 |
| Pure_G | 1.0000 | 0.0000 | 0.0000 | 0.0000 |

**Supplementary Table S5: List of all samples collected from suspected hybrids. The table includes sample id, sample type, population, sex, number of missing loci and the proportion of goat alleles (P goat) for the samples that passed the missingness filter. Last column shows which individuals were domestic (born in captivity). Samples are ordered by sample type and missingness.**

| Sample id | Sample type | Population | Sex | N.missing loci | P goat | Note ind. | Phenotypic trait with anomalies |
| --- | --- | --- | --- | --- | --- | --- | --- |
| valsusa_01 | FTA card |  | f | 63 | - | domestic | horn, body |
| queyras_16 | faeces | Val Varaita  (Queyras) | m | 63 | - |  | pelage, muzzle |
| rocciamelone_1 | faeces | Val Susa  (Rocciamelone) | m | 63 | - |  | horn, pelage, muzzle, body |
| lanzo_31 | faeces | Lanzo | m | 7 | 0.11 |  | horn, pelage, muzzle |
| lanzo_30 | faeces | Lanzo | f | 13 | 0.00 |  | horn, pelage |
| lanzo_29 | faeces | Lanzo | f | 19 | 0.26 |  | horn, muzzle, body |
| lanzo_36 | faeces | Lanzo | f | 26 | 0.47 |  | horn, pelage, muzzle, body |
| lanzo_37 | faeces | Lanzo | m | 44 | 0.11 |  | horn, pelage, muzzle, body |
| lanzo_40 | tissue |  | f | 0 | 0.80 | domestic | horn, pelage, muzzle, body |
| lanzo_32 | tissue | Lanzo | m | 0 | 0.18 |  | horn |
| lanzo_33 | tissue | Lanzo | f | 0 | 0.52 |  | horn, pelage, muzzle, body |
| lanzo_34 | tissue | Lanzo | f | 0 | 0.78 |  | horn, pelage, muzzle, body |
| lanzo_35 | tissue | Lanzo | m | 0 | 0.42 |  | horn, pelage, muzzle, body |
| ticino_1 | tissue |  | f | 1 | 0.49 | domestic | horn, pelage, muzzle, body |
| granparadiso_43 | tissue | Gran Paradiso | m | 1 | 0.49 |  | horn, pelage, muzzle |
| graubunden_1 | tissue | Graubunden | NA | 2 | 0.00 |  | pelage |
| fribourg_1 | tissue | Fribourg | m | 2 | 0.00 |  | pelage |
| granparadiso_41 | tissue | Gran Paradiso | m | 3 | 0.21 |  | horn, pelage |
| granparadiso_44 | tissue | Gran Paradiso | m | 6 | 0.17 |  | horn, pelage, muzzle, body |
| graubunden_2 | tissue | Graubunden | NA | 22 | 0.00 |  | pelage |

**Supplementary Table S6: List of major hybrid categories/generations (with posterior probability > 0.1) for each individual, including the corresponding posterior probability extracted from NewHybrid analysis. Hybrid category as in Figure S4, hybrid generation as in Figure 2.**

| **Sample id** | **Posterior probability** | **Hybrid category** | **Hybrid generation** |
| --- | --- | --- | --- |
| fribourg_1 | 1 | Pure_I | Pure_I |
| granparadiso_41 | 0.43682 | BxIxI | 3rd_gen |
|  | 0.3425 | BxI | 2nd_gen |
|  | 0.22065 | BxIxBxI | 4th_gen |
| granparadiso_43 | 1 | F1 | F1 |
| granparadiso_44 | 0.88213 | BxIxI | 3rd_gen |
| graubunden_1 | 1 | Pure_I | Pure_I |
| graubunden_2 | 0.99999 | Pure_I | Pure_I |
| lanzo_29 | 0.96722 | BxIxBxI | 4th_gen |
| lanzo_30 | 1 | Pure_I | Pure_I |
| lanzo_31 | 0.71947 | BxIxI | 3rd_gen |
| lanzo_31 | 0.24909 | BxIxBxI | 4th_gen |
| lanzo_32 | 0.14359 | BxI | 2nd_gen |
|  | 0.77514 | BxIxI | 3rd_gen |
| lanzo_33 | 0.82088 | F2 | 2nd_gen |
|  | 0.16624 | BxIxBxG | 4th_gen |
| lanzo_34 | 0.95832 | BxG | 2nd_gen |
| lanzo_35 | 0.99556 | BxGxI | 3rd_gen |
| lanzo_36 | 0.80915 | F2 | 2nd_gen |
|  | 0.16664 | BxIxBxG | 4th_gen |
| lanzo_37 | 0.86926 | BxIxI | 3rd_gen |
| lanzo_40 | 0.91368 | BxG | 2nd_gen |
| ticino_1 | 1 | F1 | F1 |

**Reference:**

**Brambilla, A., Bassano, B., Biebach, I., Bollmann, K., Keller, L., Toïgo, C., & von Hardenberg, A. (2020). Alpine ibex *Capra ibex* Linnaeus, 1758 (pp. 1–27).** <https://doi.org/10.1007/978-3-319-65038-8_32-1>

**Couturier, M. A. (1962). Le Bouquetin des Alpes: *Capra aegagrus ibex ibex* L. Couturier.**

**Giacometti, M., Roganti, R., De Tann, D., Stahlberger-Saitbekova, N., & Obexer-Ruff, G. (2004). Alpine ibex *Capra ibex ibex* x domestic goat *C. aegagrus domestica* hybrids in a restricted area of southern Switzerland. Wildlife Biology, 10(2), 137–143.** <https://doi.org/10.2981/wlb.2004.018>

**Moroni, B., Brambilla, A., Rossi, L., Meneguz, P. G., Bassano, B., & Tizzani, P. (2022). Hybridization between Alpine ibex and domestic goat in the Alps: a sporadic and localized phenomenon? Animals, 12(6). https://doi.org/10.3390/ani12060751**
